# Impacts of Protein and Energy Levels on Rumen Fermentation and Microbial Activity Under Different Incubation Temperatures

**DOI:** 10.3390/ani14213093

**Published:** 2024-10-26

**Authors:** Yong-Ho Jo, Won-Seob Kim, Yoo-Rae Kim, Mun-Su Ju, Jalil Ghassemi Nejad, Hong-Gu Lee

**Affiliations:** 1 Department of Animal Science and Technology, Sanghuh College of Life Sciences, Konkuk University, Seoul 05029, Republic of Korea; jyh3977@naver.com (Y.-H.J.); kws9285@konkuk.ac.kr (W.-S.K.); yooraekim95@gmail.com (Y.-R.K.); ju2139@naver.com (M.-S.J.); jalilghasseminejad@gmail.com (J.G.N.); 2 IANS Co., Ltd., Cheonan-si 31090, Republic of Korea

**Keywords:** energy, incubation temperatures, protein, rumen fermentation properties

## Abstract

This study aimed to explore the effects of different incubation temperatures on ruminal fermentation and rumen microorganisms and determine the appropriate protein and energy levels to enhance microbial protein synthesis using an in vitro system. Rumen inoculum was collected from two fistulated Holstein heifers (trial 1: BW: 652.3 kg ± 25.2; trial 2: BW: 683.3 kg ± 30.2) and assessed using a closed-batch culture system. The experimental model employed a 2 × 5 factorial arrangement using incubation temperatures set to 39 and 41 °C, with protein levels set to 12.0, 13.5, 15.0, 16.5, and 18.0% of DM in trial 1 or with energy levels set to 2.4, 2.5, 2.6, 2.7, and 2.8 Mcal/kg of DM in trial 2. The data were analyzed using the MIXED procedure. The results showed increased (*p* < 0.05) NH_3_-N concentrations and total volatile fatty acids (TVFAs) with higher incubation temperatures, while the liquid-associated bacterial (LAB) amounts decreased (*p* < 0.05) in trials 1 and 2. The interaction between the energy level and incubation temperature affected (*p* < 0.05) the LAB protein levels in trial 2. Higher protein levels led to increased (*p* < 0.05) NH_3_-N and acetate concentrations, but it decreased (*p* < 0.05) the propionate percentage. Conversely, higher energy levels decreased (*p* < 0.05) the amount of acetate and increased the propionate concentration, altering the acetate-to-propionate ratio. However, no interaction involving TVFA and LAB was observed between the incubation temperature and the protein or energy levels. Changes in the NH_3_-N, TVFAs, and LAB protein amounts were observed under different incubation temperatures and energy levels. In conclusion, these findings provide insight into the metabolic adaptation under different ruminal temperatures and the impacts of dietary adjustments on rumen fermentation and microbial activity. However, there are limitations to replicating the complex physiological responses that occur within the whole body solely through in vitro experiments.

## Simple Summary

This study explored the effects of protein and energy levels on rumen fermentation under different incubation temperatures using an in vitro system. We found that higher incubation temperatures increased NH_3_-N and total volatile fatty acids (TVFA) but decreased liquid-associated bacteria (LAB). Conversely, higher protein levels elevated NH_3_-N and acetate levels but reduced propionate, while higher energy levels had the opposite effect on rumen fermentation properties. In addition, incubation temperatures and energy levels were affected on rumen fermentation characteristics and LAB protein amounts. However, there were no significant interactions between energy or protein levels and incubation temperatures for TVFA and LAB. The findings suggested that the adjustment of single nutrient levels of protein or energy would not be sufficient to enhance microbial protein synthesis under different incubation temperatures.

## 1. Introduction

External environmental temperature is closely related to the ruminal microbial ecosystem in cattle [1,2,3]. Cows reduce their feed intake and choose feeds with less fermentation heat to prevent an increase in body temperature under high-ambient-temperature conditions [2,4,5]. Ruminal and core temperature are also influenced by the external environment and type of diet [6,7]. This results in a decrease in ruminal fluid pH, leading to abnormal fermentation characteristics. Consequently, ruminal bacteria are reduced [1,2]; this process is closely correlated to decreasing amounts of microbial proteins. Microbial proteins make up an average of 59% of the total metabolic protein [8]. For microbial protein synthesis, the amount of digestible organic matter (OM) and the balance of energy and protein are important factors [8,9]. As previously mentioned, cows decrease their OM intake under high-ambient-temperature conditions, a mechanism that is not easy to prevent through nutritional manipulation. To accomplish an appropriate balance between energy and protein, it is necessary to consider not only the energy or protein content but also the rate of degradation. The Cornell Net Carbohydrate and Protein System is used for classifying proteins into fractions A, B1, B2, B3, and C [10]. In addition, energy values can be calculated according to the NRC [11] equation, and neutral detergent fiber (NDF), non-fiber carbohydrate (NFC), and crude fat are adjusted when the energy is increased. Since rumen fermentation heat promotes the intake of certain feed ingredients, during the summer, the consumption of concentrated feed becomes more crucial than that of forage-based feed [12]. The intake of concentrated feed increases the fermentation rate, leading to a drop in pH, especially during summer, and it results in a lower acetate-to-propionate ratio. During the summer season, both intake and rumination times are reduced. Additionally, the reduction in chewing stimulation decreases the saliva immersion ratio.

Numerous studies demonstrate the impacts of varying energy and protein levels on microbial protein synthesis under optimal fermentation temperatures [13,14]. However, these previous studies have limitations because using animals to validate the impacts of external environmental temperature on rumen fermentation properties overlooks the decrease in DMI and the physiological adaptations these animals make to maintain their balance. Thus, in vitro models for evaluating different incubation temperatures should incorporate varied nutrient levels. To the best of our knowledge, there is no existing study investigating the relationship between the quantities of microbial proteins and the nutrient compositions under different incubation temperatures. To address this knowledge gap, we hypothesize that the compositions of protein and energy levels in a diet influence rumen fermentation properties, and optimizing these diet levels can improve rumen fermentation under different incubation temperatures in ruminants.

We conducted two distinct trials to investigate these hypotheses. The objective of trial 1 was to determine the appropriate protein level while maintaining consistent levels of energy components such as NFC and NDF, subsequently increasing the protein content based on its degradation rate. In trial 2, the objectives were to identify the suitable energy level while maintaining the protein composition constant and to increase the energy levels by raising the NFC and reducing the NDF. Additionally, we aimed to confirm the effects of different incubation temperatures on ruminant microorganisms independent of the physiological response of the host.

## 2. Materials and Methods

All the procedures involving animals were approved by the Institutional Animal Care and Use Committee (IACUC) of Konkuk University (Approval No: KU21011) (Seoul, Republic of Korea).

### 2.1. Experiment Design, Collecting Ruminal Inoculum and In Vitro Incubation Procedures

Two rumen-fistulated Holstein heifers (trial 1 BW: 652.3 kg, SD: 25.2; trial 2: BW: 683.3 kg, SD: 30.2) were utilized to acquire rumen fluid samples. These cows were fed a 6:4 ratio of forage to concentrate for 2 weeks prior to sampling, according to the maintenance requirements outlined by the NRC [11], with water and minerals available ad libitum. The experimental model employed a 2 × 5 factorial arrangement using incubation temperatures of 39 and 41 °C with 5 protein levels (12.0, 13.5, 15, 16.5, and 18%) or 5 energy levels (2.4, 2.5, 2.6, 2.7, and 2.8 Mcal/kg) for each trial. Rumen fluid was collected from the ventral and dorsal sacs of two heifers 2 h prior to morning feeding during a thermoneutral period. The temperature–humidity index remained consistently below 70, which is recognized as the upper critical threshold for cattle [15]. The collected rumen fluid was then promptly transported to a laboratory within 20 min of collecting using preheated thermos bottles. Subsequently, the rumen fluid was filtered through a nylon filter with a pore size of 50 µm. Finally, the rumen fluid collected from the two heifers was mixed in equal proportions.

The mixed rumen fluid was prepared by combining Menke’s buffer and rumen fluid in a ratio of 1:3 (*v*/*v*) [16]. The inoculum was heated at 39 °C and continuously purged with CO_2_. Menke’s buffer consisted of 9.52 mM Na_2_PO_4_, 10.8 mM KH_2_PO_4_, 0.58 mM MgSO_4_·7H_2_O, 1.08 µmol CaCl_2_·2H_2_O, 0.61 µmol MnCl_2_·4H_2_O, 0.05 µmol CoCl_2_·6H_2_O, 0.05 µmol FeCl_2_·4H_2_O, 8.66 mM NH_4_HCO_3_, 71.3 mM NaHCO_3_, 0.05 µmol Resazurin sodium salt, 147 µmol NaOH, and 2.97 µmol Na_2_S·9H_2_O.

The substrates were ground through a 2-mm screen and used as a substrate for incubation. Then, 30 mL of buffered rumen fluid was added to 50 mL serum bottles containing F57 filter bags (pore size 25 µm, Ankom Technology Corp., Macedon, NY, USA); each bag contained 0.3 g of substrate [17]. Ultra-high purity (99.99%) CO_2_ was flushed into the headspaces of the bottles, which were incubated for 3, 6, 12, 24, and 48 h during each treatment. The experiments were conducted three times, with two replications per experiment. Thus, a total of 2 (39 and 41 °C) × 5 (concentrations) × 5 (incubation time) × 3 (incubation replication) × 2 (bottle replication) × 2 (trials; protein or energy) + 60 bottles (blank bottle per each treatment) = 660 bottles were used.

Table 1 presents the results of the chemical composition based on the feed ingredient used in trial 1. In trial 1, five substrates were formulated with varying protein concentrations of 12.0, 13.5, 15, 16.5, and 18% while maintaining a constant metabolizable energy (ME) level of 2.7 (Table 2). The chemical composition of the feed ingredients used in trial 2 is presented in Table 3. In trial 2, five substrates were formulated with varying metabolizable energy concentrations of 2.4, 2.5, 2.6, 2.7, and 2.8 Mcal/kg while maintaining a constant protein content of 17.5% dry matter (DM) and considering the protein fraction (Table 4). Dietary protein and energy levels were selected based on recommendations from the eighth beef cattle NRC (2016) guidelines and findings from previous studies [5,18].

### 2.2. Chemical Analysis

For the in vitro batch culture, twelve feed ingredients were used. These ingredients were mixed to adjust the protein or energy level of the experimental substrate (Table 1 and Table 3). Each feed ingredient was dried completely for three days at 55 °C, and the crude protein (CP) was measured using the Kjeldahl automatic distiller (Kjeltec 8400 Analyzer, Hillerød, Denmark), as described by AOAC, with N × 6.25 (method 976.05) [20]. To differentiate proteins based on their digestion rate, nonprotein nitrogen (fraction A), true soluble protein (fraction B1), insoluble protein-neutral soluble detergent protein (fraction B2), and neutral insoluble detergent protein that was soluble in acid detergent (fraction B3) and insoluble in acid detergent (fraction C) were measured using the method outlined by Licitra, Hernandez and Van Soest [10]. Ether extract (EE) was measured using the Ankom XT15 extractor (Ankom Technology Corp., Macedon, NY, USA) with method 920.39, while ash (method 942.05; 550 °C) was measured using a furnace (FHX-63, Daihan Scientific Co., Wonju, Republic of Korea), as described by AOAC [20]. Neutral detergent fiber (NDF) and acid detergent fiber (ADF) were analyzed using the method described by Van Soest et al. [21] with the Ankom 200 fiber analyzer (Ankom Technology Corp., NY, USA); heat-stable α-amylase (Ankom Technology Corp., NY, USA) was used to analyze the NDF. Acid detergent lignin (ADL) was measured using the method outlined by Van Soest and Robertson [22]. Non-fiber carbohydrate (NFC) was calculated using the following equation with the chemical analysis parameters: NFC = 100 − ash − EE − (CP − NDIP) − NDF. Additionally, the equations provided by the NRC [11] were used to evaluate the energy values of the feed ingredients, expressed as total digestible nutrients (TDN) and metabolizable energy (ME).

The experimental feed of trial 1 increased the protein content while keeping the energy constant, reducing the non-fiber carbohydrates from 43.9 to 40.3%. However, as the energy value was set higher in feeds with increased protein contents, the ether extract was reduced from 2.23 to 1.04%. Consequently, the five-step protein concentration feeds used in this experiment had an ME of 2.7 Mcal/kg (Table 2).

The experimental feeds of trial 2 were designed to maintain a constant protein fraction ratio with a fixed protein content of 17.5%. As the energy levels increased, the NFC gradually rose from 39.60 to 54.62%. Conversely, with higher ME levels, the NDF progressively decreased from 38.65 to 25.0%. The EE experienced a slight increase from 1.31 to 1.86% since excessive concentrations could affect the fermentation in the rumen. As a result, the five-step ME concentration feeds used in this experiment had a CP of 17.5% (Table 4).

### 2.3. Analyses of Fermentation Properties

Following the incubation of the vials for 3, 6, 12, 24, and 48 h, the pH values were measured using a digital pH meter (S20 SevenEasy pH; Mettler Toledo Co., Ltd., Greifensee, Switzerland). Residual rumen fluid samples were then immediately stored at −20 °C for the subsequent analysis of volatile fatty acids (VFAs) and NH_3_-N. Upon thawing, 10 mL of the sample was mixed with 1 mL of HgCl_2_ 2% (*w*/*v*) and briefly centrifuged at 2000× *g* for 10 min at 4 °C to remove the feed particles. The resulting supernatants were used for VFA and NH_3_-N analysis. To prepare the samples for the VFA analysis, 1.4 mL of the supernatants was first mixed with 0.28 mL of 25% (*w*/*v*) meta-phosphoric acid and then centrifuged again at 20,000× *g* for 20 min at 4 °C. Next, 1 mL of the supernatant was mixed with 50 µL of 2% (*w*/*v*) pivalic acid as an internal standard. The VFA profile was measured using a gas chromatograph (HP 6890 series GC system; Agilent Technologies Inc., Santa Clara, CA, USA) equipped with a flame ionization detector and a capillary column (DB-FFAP; Agilent Technologies Inc., Santa Clara, CA, USA). The inlet, oven, and detector temperatures were set to 220, 100, and 250 °C, respectively. Each sample for VFA analysis was duplicated three times.

To determine the NH_3_-N concentration, the centrifuged samples were subjected to catalyzed indophenol reactions [23] and analyzed using spectrophotometry (Synergy2; Biotek Instruments, Inc., Winooski, VT, USA). In brief, NH_3_-N standards and samples (2 µL) were mixed with 147 µL of phenol color reagent (531 mM phenol, 0.95 mM sodium nitroferricyanide) and 125 µL of alkali-hypochlorite (625 mM sodium hydroxide, 28.2 mM sodium hypochlorite) in a 96-well cell plate. The reaction was carried out in a 55 °C dry oven for 10 min, followed by the measurement of absorbance at 630 nm using spectrophotometry. Each sample was triplicated and analyzed three times (3 × 3).

### 2.4. Protein Analysis of Liquid-Associated Bacteria (LAB)

The residual rumen fluid intended for LAB analysis was stored at −4 °C and examined using a bicinchoninic acid (BCA) protein assay kit (Thermo Fisher, Waltham, MA, USA). Prior to measuring the microbial protein content, the pretreatment method outlined by Makkar et al. [24] was followed. The stored rumen fluid was thawed and vortexed at 3000 rpm for 10 s to gently detach the microbes from the substrate or the samples. Subsequently, it was centrifuged at 400× *g* for 5 min to eliminate feed and protozoa. Next, 1 mL of supernatant was transferred and centrifuged at 25,000× *g* for 20 min. The supernatant was removed, leaving only the pellet at the bottom of the tubes. Then, 1 mL of deionized water was added, and the mixture was vortexed at 3000 rpm for 10 s, followed by another centrifugation at 25,000× *g* for 20 min. After discarding the supernatant, 1 mL of 0.25 N NaOH was added and vortexed at 3000 rpm for 10 s. The mixture was digested using a heating machine at 100 °C for 10 min before being centrifuged at 25,000× *g* for 30 min. Lastly, 25 µL of the supernatant was transferred onto a 96-well plate. The total protein content in the supernatant was determined using the bicinchoninic acid (BCA) assay (Thermo Fisher Scientific, Waltham, MA, USA) with spectrophotometry (Synergy 2, Biotek Instruments Inc., Santa Clara, CA, USA), measuring the absorbance at 562 nm.

### 2.5. Statistical Analysis

The fermentation properties and LAB data were analyzed using the MIXED procedure in SAS version 9.4 (SAS Institute Inc., Cary, NC, USA). The model used was as follows:

Y_ijk_ = µ +α_i_ + β_j_ + αβ_ij_ + γ_k_ + ε_ijk,_ (1)

where Y*_ijk_* represents the observation of incubation run *k* for the given treatments *i* and *j*, µ is the overall mean, α*_i_* is the fixed effect of incubation temperature *i* (39 and 41 °C), β*_j_* is the fixed effect of the protein or energy level of substrate *j* (CP 12.0, 13.5, 15.0, 16.5, and 18.0% of DM or ME 2.4, 2.5, 2.6, 2.7, and 2.8 Mcal/kg), αβ*_ij_* is the interaction between the incubation temperature and the protein or energy level of the substrate, γ*_k_* is the random effect of incubation run *k* nested in treatments *i* and *j*, and *ε_ij_* is the residual effect. The data were presented as least squares means with the standard error of the mean. When a treatment indicated a significant effect, the least squares means were compared using Tukey’s post hoc comparison. Differences were considered statistically significant if the *p*-value was less than 0.05. If the *p*-value was greater than 0.05, it indicated that there was no statistically significant difference.

## 3. Results

### 3.1. In Vitro Fermentation Properties

In trial 1, the results indicated that there were significant changes in the pH and NH_3_-N concentrations as well as in the TVFA compositions with increasing fermentation temperatures. The pH of the inoculum was significantly higher at 41 °C after 3 h of fermentation (*p* < 0.001, Table 5), but there was no significant difference in the pH after that time. On the other hand, the NH_3_-N concentration was found to be significantly higher in the 41 °C fermentation environment compared to the 39 °C fermentation environment after 3, 12, 24, and 48 h. In terms of VFA composition, it was found that the concentration of *iso*-butyrate and *iso*-valerate gradually increased at a fermentation temperature of 41 °C. The concentration of *iso*-butyrate increased to about 6, 7, 11, and 13% after 6, 12, 24, and 48 h, respectively, while the concentration of *iso*-valerate gradually increased to about 6, 14, and 16% after 12, 24, and 48 h compared to the 39 °C fermentation environment. It was confirmed that, as the incubation temperature increased to 41 °C, the TVFAs also increased after 3, 6, and 12 h (*p* < 0.05, Table 5). In particular, the TVFA levels rose sharply by approximately 30% within the first 3 h. However, after 48 h incubated at 41 °C, the TVFA levels decreased by about 19.14% from 104.6 to 84.6 mM (*p* = 0.01, Table 5). Additionally, analyzing the change in VFA composition under 41 °C incubation temperatures for 48 h revealed a slight reduction in the acetate concentration, while the butyrate, *iso*-butyric acid, valerate, and *iso*-valerate concentrations all increased (*p* < 0.05).

In trial 2, upon increasing the incubation temperature from 39 to 41 °C, significant differences in the NH_3_-N, TVFA, and LAB were observed (*p* < 0.01, Table 6). The NH_3_-N levels were 19.7, 10.6, 12.4, and 30.9% higher at fermentation times of 3, 6, 12, and 24 h (*p* < 0.001). Compared to an incubation temperature of 39 °C, the TVFA levels increased by 13.5, 10.6, and 11.1% after 3, 6, and 12 h when the temperature was 41 °C (*p* < 0.01), with no significant differences after 24 and 48 h of fermentation.

### 3.2. In Vitro Fermentation Properties According to the Protein Levels of the Trial 1 Diet

The concentration of NH_3_-N was consistent at protein levels of 12.0, 13.5, and 15.0%, but significantly increased at 16.5 and 18.0% CPs (*p* < 0.05, Table 5). After 3 h of fermentation, the concentration of generated NH_3_-N increased by 8.9% and 11.6% for CPs of 16.5% and 18.5%, respectively, compared to a CP of 12.0% (Table 5, *p* = 0.001). Throughout all the fermentation times, the NH_3_-N levels remained the highest for CPs of 16.5 and 18.0%, with a particularly large difference in concentration of 41.7–50.9% after 12 h of fermentation. The difference decreased to about 10.9% after 24 h of fermentation. Additionally, the increase in acetate was found to be significant (*p* < 0.01) with an increasing protein content after 12 h of fermentation, while the propionate decreased from CPs of 16.5 and 18.0% after 24 h of fermentation compared to a protein content of 12.0%. Valerate was also found to increase by approximately 24.1% over a 6 h fermentation period when using CPs of 16.5 and 18.0% (Table 5).

### 3.3. In Vitro Fermentation Properties According to the Energy Levels of the Trial 2 Diet

Changes in the energy level influenced the pH, acetate, propionate, butyrate, TVFA, and the ratio of acetate to propionate (*p* < 0.01, Table 6). After 12, 24, and 48 h of fermentation, the pH decreased as the energy level increased (*p* < 0.001). The acetate concentrations declined when the energy level exceeded 2.6 Mcal/kg, with higher energy levels resulting in lower acetate production rates (*p* < 0.001). Conversely, the propionate concentrations increased, displaying an inverse relationship with the production ratio pattern of acetate (*p* < 0.001). Thus, the ratio of acetate to propionate was found to decrease with increasing energy levels. The production rates of butyrate increased linearly as the energy level rose. Based on an energy level of 2.4 Mcal/kg after 24 h of fermentation, the increases in the production ratios of butyrate were 1.3, 9.2, 16.1, and 21.8% at 2.5, 2.6, 2.7, and 2.8 Mcal/kg, respectively. The TVFA concentrations did not show significant differences even when the energy level increased to 2.6, 2.7, and 2.8 Mcal/kg after 24 h of fermentation and up to 2.5 Mcal/kg after 48 h. Increasing the energy level above 2.5 Mcal/kg while maintaining a constant protein content of 17.5% did not result in further increases in the TVFA concentrations. The BCFA, valerate, and protein amounts in LAB were not affected by the energy levels (Table 6).

After 3 h of fermentation, the pH exhibited a decreasing pattern with an increase in the energy level, but, at 41 °C, the pH increased (*p* = 0.011). NH_3_-N also displayed different patterns between 39 and 41 °C: the NH_3_-N increased as the energy levels rose at 39 °C but decreased at 41 °C (*p* = 0.009). The TVFA concentration increased at 2.6 and 2.7 Mcal/kg at 41 °C compared to 39 °C after 6 h of fermentation (*p* = 0.002). Regarding the VFA composition, a significant difference was observed between a decrease in acetate and an increase in propionate at 2.7 Mcal/kg at 41 °C (*p* < 0.05) resulting in a reduced A:P ratio (*p* = 0.025). After 12 h of fermentation, the NH_3_-N concentration was higher at 41 °C for ME 2.4 and 2.6 Mcal/kg compared to 39 °C, and in the case of TVFA, increases were shown at 41 °C for ME 2.4, 2.6, and 2.7 Mcal/kg treatments compared to 39 °C (*p* = 0.006). However, after 24 and 48 h of fermentation, no interaction was found between the energy levels and the incubation temperatures in the fermentation properties.

### 3.4. Changes in Protein Amount of LAB

In trial 1, during fermentation at an incubation temperature of 41 °C for 6 h, the protein of LAB decreased by 6% compared to 256 µg/mL at 39 °C, reaching 240.8 µg/mL (*p* < 0.001, Table 5). While there was no significant difference after 12 h of culture due to high variations, by 24 h, the protein level decreased by approximately 24% to 149.4 µg/mL at 41 °C compared to the value of 196.7 µg/mL at 39 °C (*p* < 0.001, Table 5).

In trial 2, the protein amounts in LAB initially increased by 13.4 and 11.0% after 3 and 6 h, respectively, at a temperature of 41 °C, but they decreased by 33.3% after 48 h of fermentation (Table 6). The protein amounts in LAB were not affected by the energy levels. An interaction between incubation temperatures and the energy level was observed after 48 h of fermentation (*p* < 0.001): the protein amounts in LAB increased as the energy level rose at an incubation temperature of 39 °C; however, under 41 °C incubation conditions, the protein amounts in LAB remained approximately 52.1 µg/mL lower than those at 39 °C despite the increase in the energy level.

## 4. Discussion

### 4.1. In Vitro Fermentation Properties

In an in vitro experiment, it should be noted that protozoa and fungi exhibit reduced activity and have lower absorption and secretion rates within rumen epithelial cells. However, in vitro experiments like the one conducted in this study maintain constant substrate levels corresponding to the feed intake and consistent incubation times, representing the passage rates. This methodology allows for the examination of the direct effects of different incubation temperatures on rumen microorganisms while excluding other confounding factors.

A previous study reported that the reticular temperature exceeded 40.5 °C in dairy cows exposed to high ambient temperatures [4]. They also observed a correlation between reticular temperature and core temperature under high-ambient-temperature conditions. In this study, the incubation temperature was set based on the positive correlation between the highest rumen reticular temperature and an incubation temperature close to 41 °C [4]. The experiment was designed to expose rumen microorganisms to high ambient temperatures.

It is commonly observed that the pH inside the rumen often decreases during heat stress experiments with cattle [25]. In our current study, in trials 1 and 2, incubation temperatures did not induce a decrease in the pH. Conversely, C King et al. [26] reported that an increase in the incubation temperature resulted in a higher pH. It is believed that the pH reduction associated with ruminal temperature does not have a direct effect on ruminal microorganisms but rather a response to the host experiencing high ambient temperatures. Factors contributing to a decreased pH include changes in DMI, a high-concentrate diet, reduced saliva secretion, and alterations in the digestive system’s mobility and absorption [27,28,29]. However, we cultured only rumen microorganisms in vitro and found that the pH did not decrease when high incubation temperature was applied directly to these microorganisms. While the exact reason for the stability of the pH remains unclear, this suggests that the decrease in ruminal pH during the summer season in vivo may be attributed not to the microorganisms themselves but rather to the animal’s homeostatic responses or behavioral changes.

The increase in TVFA during fermentation (3–12 h) at 41 °C observed in this study was consistent with the findings of trial 1, where an increase in TVFA was observed for up to 12 h of fermentation. The optimal temperature for rumen microorganisms is unknown, as the rumen-reticular temperature varies from 38 to 40 °C and consists of an ecosystem of over 200 bacterial species, more than 25 species of protozoa, and five types of fungi [30]. Each microbial species has a unique optimal temperature at which the maximum growth rate and activity are achieved. For instance, *Saccharomyces cerevisiae*’s optimal incubation temperature is 40.9 °C [31], while that of *Ruminococcus albus* is 44.0 °C [32]. Although the kinds of microbial species in this study that would be activated at the 41 °C incubation temperature are still unknown, combining the findings of trial 1 and trial 2 confirmed that the TVFA concentration in ruminants increased at 41 °C until the 12 h mark, suggesting that certain microbial species may become more active under high-ambient-temperature conditions.

Additionally, the *iso*-butyrate and *iso*-valerate concentrations increased by approximately 6–16% after 12 h of fermentation in trial 1. These two VFAs are branched-chain fatty acids (BCFAs) that form when amino acids such as valine, isoleucine, leucine, and proline are used as precursors during decomposition. Although the association between BCFAs and high incubation temperature has not been extensively studied, fibrolytic bacteria require BCFAs for growth and proliferation [33,34]. The increase in BCFAs is thought to be a result of reduced BCFA usage for synthesis due to decreased microbial protein synthesis from NH_3_-N under high incubation temperature, causing a relative increase in BCFA concentrations.

The results of trial 1, trial 2, and C King et al. [26] indicate that the changes in the proportions and amounts of acetate, propionate, butyrate, and valerate observed under high-ambient-temperature conditions in animals may be due to physiological adjustments to maintain homeostasis rather than being a direct result of microbial activity.

During the initial stage of fermentation, including the period from 3 to 12 h, an upward trend in the protein amounts of LAB was observed, accompanied by an increase in TVFA at an incubation temperature of 41 °C. These findings suggest an elevation in the NH_3_-N level that can be attributed to the augmented degradation rate of the substrates. However, after 12 h of fermentation, declines in both the TVFA and protein amounts of LAB were observed under high incubation temperatures. This showed that prolonged high incubation temperatures adversely affected microbial activity, resulting in reduced microbial synthesis when utilizing NH_3_-N [35,36]. The observed decreases in the protein amounts in LAB under high incubation temperatures further supported the notion of reduced microbial protein synthesis. Specifically, the protein amounts of LAB decreased by 33.3% after 48 h of fermentation in trial 2, suggesting the significant impact of high incubation temperature on rumen microorganisms.

### 4.2. In Vitro Fermentation Properties According to the Protein Levels of the Trial 1 Diet

When the CP levels were at 16.5 and 18.0%, NH_3_-N was increased after 3 h of fermentation. This occurred because, as protein levels rose, the NPN also increased to maintain the same protein fraction ratio. Additionally, after 12 h of fermentation, the acetate levels in treatments with CPs of 16.5 and 18.0% rose by approximately 21% compared to those with a CP of 12.0%. This was believed to result from a reduction in the NFC content even though the NDF content remained similar. After 24 h, propionate concentration declined by around 15.5% in treatments with CPs of 16.5 and 18.0% compared to a CP of 12.0%. This was considered to be the reverse of the acetate increase observed [37]. This delay in fermentation may be attributed to the fact that acetate production was at 2.81 moles/12 h, while propionate production was at a slower rate of 0.82 moles/12 h, as previously reported [38]

Valerate production after 6 h of fermentation in treatments with CPs of 16.5 and 18.0% increased by approximately 14.1% compared to a CP of 12.0%. Branched-chain amino acids, including leucine and isoleucine, are precursors in valerate production; thus, an increase in protein content enhances the valerate levels [39]. Moreover, when the ratio of soluble protein to a CP of 14.0% increases to 20–50% (equivalent to a CP of 2.8 to 7% of DM basis), the valerate amounts increase in conjunction with 30% or more soluble protein content [40]. In this study, since all the protein types increased with the rising CP levels, this could be attributed to the increase in soluble protein.

Significant differences were observed in the fermentation properties and LAB between the incubation temperatures and protein levels. The results indicated that while protein levels significantly influenced the ruminal fermentation properties, no interaction was observed between protein levels and incubation temperatures. Thus, it might not be necessary to consider protein levels when evaluating ruminal fermentation properties and the quantity of microbial protein synthesis under high-ambient-temperature conditions.

### 4.3. In Vitro Fermentation Properties According to the Energy Levels of the Trial 2 Diet

When investigating the effects of high temperatures on rumen fermentation properties using animals, the direct impact was that the pH decreased after 12 h of fermentation when using ME 2.6, 2.7, and 2.8 Mcal/kg. The increase in NFC and the decrease in NDF in the diet are potential reasons for this effect [41,42]. In the current study, a level of energy setting of at least 5 levels was required to statistically determine the adequate level of energy. When the concentration was set to increase linearly, it reached an ME of 2.8 Mcal/kg. With the ingredient used in this study, setting the energy concentration higher than this led to extreme changes in the ratio of concentrate and roughage. The objective of this study was to gradually increase the energy requirement from 2.4 Mcal/kg while maintaining constant NFC and NDF contents. For this reason, although decreasing the NDF content at the ME 2.8 Mcal/kg level could potentially cause ruminal acidosis when fed to cows, it was challenging to consider the impact on cows when determining the energy content.

Acetate is indeed one of the primary VFAs produced during ruminal fermentation, and it is generated in the highest proportion compared to other VFAs like propionate and butyrate. Acetate-producing bacteria play a vital role in the process of converting one molecule of pyruvate and one molecule of H_2_O into one molecule of acetate, one molecule of CO_2_, and two hydrogen atoms [43]. In this study, the production ratio of acetate decreased with increasing energy levels starting from 6 h of fermentation. This indicated that increasing the energy level in the fermentation resulted in a linear decrease in the production ratio of acetate, with the highest reduction observed at ME 2.8 Mcal/kg. This decrease in acetate concentration could be attributed to the increased availability of NFC and the decreased availability of NDF in the substrate. It is important to note that acetate is mainly synthesized from slowly digestible fiber sources, such as NDF, which has a degradation rate of 0.03–0.09/h [44]. Therefore, the linear decrease in the acetate production ratio after 48 h of fermentation with increasing energy levels could have been due to the higher availability of rapidly fermentable carbohydrates and the lower availability of slowly fermentable NDF when using higher energy levels, especially compared to ME 2.4 Mcal/kg, which contained more NDF.

Propionate-producing bacteria could convert one molecule of pyruvate and four hydrogen atoms into one molecule of propionate and one molecule of H_2_O through two different pathways: the succinate and acrylate pathways [43]. Starch-rich diets have been shown to support the development of propionate-producing bacterial species and are associated with an increase in the proportion of propionate, as reported by Makkar and McSweeney [30]. The production ratio of propionate increased with increasing energy levels starting from 6 h of fermentation. After 24 h of fermentation, the production ratio of propionate increased by 0.9, 12.2, 14.1, and 12.9 when ME was increased to 2.5, 2.6, 2.7, and 2.8 Mcal/kg of DM compared to 2.4 Mcal/kg, respectively. Similarly, after 48 h of fermentation, the increase in the production ratio of propionate was 0.9, 5.5, 10.5, and 12.1% for ME levels of 2.5, 2.6, 2.7, and 2.8 Mcal/kg of DM, respectively. The rapid digestion of NFC, which had a degradation rate of 0.05 to 0.50/h, may explain the increase in propionate concentration within a 24 h period [44,45]. The decrease in acetate and increase in propionate led to a decrease in the A:P ratio after 6 h of fermentation, with reductions of 7.1, 17.0, 18.8, and 19.2% when ME was increased to 2.5, 2.6, 2.7, and 2.8 Mcal/kg of DM compared to 2.4 Mcal/kg, respectively. The levels of NFC and reduced NDF could have influenced the concentration of acetate, propionate, and the A:P ratio [41,42].

The study by Dijkstra, Forbes and France [43] explains the process of transforming two molecules of pyruvate into one molecule of butyrate and two molecules of CO_2_. The findings of this study showed that the butyrate production rate increased as the energy level increased during fermentation, particularly after 6 h and continuing linearly over 24 and 48 h of fermentation. The observed changes in the C4 production rates could potentially alter the population of butyrate-producing bacteria, such as *Butyrivibrio fibrisolvens* or large protozoa. This idea was supported by Russell and Baldwin [46] and Williams and Coleman [47], who suggested that an increase in energy levels may lead to changes in the microbial population. This finding is important because it provides a possible explanation for the observed increase in butyrate concentration as the energy levels increase during fermentation. Overall, this study highlights the relationship between energy levels and the concentration of butyrate, as well as the potential effects on microbial populations. Additional research is warranted to gain a deeper understanding of the underlying mechanisms driving these relationships and to explore how microbial populations can be enhanced to improve the efficiency of fermentation processes.

The results of the study showed that treatment with ME levels above 2.6 Mcal/kg increased TVFA, leading to the conclusion that ME levels above 2.6 Mcal/kg are sufficient to enhance TVFA synthesis in the rumen. After 48 h of fermentation, the interactions between the incubation temperatures and the energy levels were evident in the protein amount of LAB. In the treatments with ME 2.6 and 2.8 Mcal/kg, an increase in the protein amount of LAB was observed at 39 °C, but not at 41 °C. Despite controlling the energy level, a high incubation temperature still caused a reduction in the protein amounts of LAB.

## 5. Conclusions

The rumen environment is influenced by multiple mechanisms. In this in vitro experiment, the direct effects of different incubation temperature on microbial activity were examined. In conclusion, this study found that increased ruminal temperature elevated the NH_3_-N content and reduced the TVFA and VFA concentrations due to decreased microbial protein synthesis. Varying protein and energy levels showed distinct effects. Higher protein levels raised the NH_3_-N, acetate, valerate, and BCFA concentrations while lowering propionate levels, whereas increased energy levels led to lower pH and acetate levels and higher propionate, butyrate, and LAB protein levels. These findings provide insight into metabolic adaptation under different ruminal temperatures and the impacts of dietary adjustments on rumen fermentation and microbial activity.

However, given the findings of this study, adjusting the protein and energy levels may not be necessary to improve rumen fermentation and microbial protein synthesis under high-incubation-temperature conditions. It should be noted that the results of this study are based on in vitro experiments, which may not fully reflect the physiological response of the host and the complex interactions surrounding ruminant microorganisms. Therefore, further study is needed to confirm the changes in microbial protein synthesis under different temperature conditions, as well as alterations in amino acid composition resulting from shifts in the microbial composition using cattle.

## Figures and Tables

**Table 1:** The chemical compositions of the feed ingredients used in trial 1.

Items (% of DM) ^1^	Corn Grain, Ground, Dry	Wheat	Wheat Bran	Soybean Meal	Distillers Grains with Soluble, Dry	Rapeseed Meal	Sesame Meal	Corn Gluten Feed, Dry	Corn Gluten Meal, Dry	Rice Straw, Mature	Tall-Fescue Hay, Mature	Alfalfa, Hay
CP	7.84	12.46	16.48	48.48	29.10	37.80	42.50	19.40	66.40	3.73	5.67	15.10
A	0.30	1.37	1.79	3.01	1.93	5.75	3.64	8.07	2.04	0.00	0.00	1.07
B1	0.86	1.53	2.43	1.12	1.39	6.78	2.47	2.95	2.01	0.98	0.92	0.22
B2	5.55	6.47	7.73	40.76	16.91	17.65	21.01	5.85	53.73	0.63	2.92	10.82
B3	0.99	2.98	4.12	3.43	8.10	0.00	1.90	2.05	8.62	1.43	0.49	2.42
C	0.14	0.11	0.41	0.16	0.77	7.62	13.48	0.48	0.00	0.69	1.34	0.57
NDIP	1.13	3.09	4.52	3.59	8.87	7.43	15.38	2.53	8.62	2.12	1.83	2.99
ADIP	0.14	0.11	0.41	0.16	0.77	7.62	13.48	0.48	0.00	0.69	1.34	0.57
NDF	11.15	15.87	41.23	15.30	48.13	32.14	42.87	43.19	12.10	69.51	71.07	35.42
ADF	2.53	3.65	12.22	6.75	13.72	28.99	36.22	21.30	2.68	36.74	41.01	24.94
ADL	0.41	0.39	3.04	0.59	1.38	16.93	20.12	2.97	1.05	4.58	5.06	4.70
EE	3.21	0.84	3.25	0.77	7.68	0.61	13.40	0.99	1.49	1.03	0.62	1.33
NFC	77.68	72.16	38.55	32.30	18.43	27.52	8.51	33.25	24.69	16.26	14.27	41.94
Ash	1.25	1.77	5.02	6.75	5.51	9.38	8.13	5.72	3.95	11.6	10.2	9.20
TDN ^2^	88.23	87.33	67.56	80.27	80.27	55.99	67.35	68.10	85.69	51.27	51.60	63.86
tdNFC	76.13	73.55	37.78	31.66	18.06	26.97	8.34	32.59	24.20	15.93	13.98	41.11
tdCP	7.79	12.41	16.31	48.41	28.82	34.73	37.08	19.18	66.39	2.99	4.27	14.43
tdFA	2.21	0.00	2.25	0.00	6.68	0.00	12.40	0.00	0.49	0.03	0.00	0.33
tdNDF	6.35	8.38	20.46	7.20	25.36	1.30	1.04	23.33	1.00	39.27	40.35	14.59
DE ^3^	3.81	3.84	3.27	4.04	3.77	2.83	3.34	3.12	4.52	2.19	2.22	2.88
ME ^4^	3.40	3.42	2.85	3.63	3.37	2.41	2.97	2.70	4.12	1.76	1.79	2.46

^1^ CP: Crude protein; A: protein A fraction represents the soluble nonprotein N. Degradable rate in rumen is direct; B1: protein B1 fraction is the soluble true protein. Degradable rate in rumen is 120–400%/h; B2: protein B2 fraction represents protein with intermediate rates of degradation. Degradable rate in rumen is 3–16%/h; B3: protein B3 fraction is the CP insoluble in neutral detergent solution but soluble in acid detergent solution. Degradable rate in rumen is 0.06–0.55%/h; C: protein C fraction is the unavailable nitrogen [10,19]; NDIP: CP insoluble in neutral detergent solution; ADIP: CP insoluble in acid detergent solution; NDF: neutral detergent fiber; ADF: acid detergent fiber; ADL: acid detergent lignin; EE: ether extract; NFC: non-fiber carbohydrates. ^2^ True digestibility nutrition (%) = true digestibility non-fiber carbohydrate (tdNFC) + true digestibility crude protein (tdCP) + true digestibility fatty acid (tdFA) × 2.25 + true digestibility neutral detergent fiber (tdNDF) − 7 [11]. ^3^ Digestible energy (DE) = (tdNFC/100) × 4.2 + (tdCP/100) × 5.6 + (tdFA/100) × 9.4 − 0.3) [11]. ^4^ If EE was lower than 3%, metabolizable energy (ME) was (1.01 × DE) − 0.45, if EE was higher than 3%, ME was ((1.01 × DE) − 0.45) + 0.0046 × (EE − 3) [11].

**Table 2:** The feed ingredients and chemical compositions used in trial 1.

Ingredient (%)	CP 12.0%	CP 13.5%	CP 15.0%	CP 16.5%	CP 18.0%
Corn grain, ground, dry	33.81	32.10	22.33		
Wheat			7.29	29.45	26.31
Soybean Meal, solvent, 44% CP	3.86				
Distiller grains with soluble, dried	7.22	2.78	4.16		
Rapeseed meal			1.11	2.37	4.66
Sesame meal		1.59	1.48	0.07	
Corn gluten feed, dry	1.10	1.98		0.01	0.17
Corn gluten meal, dry		6.53	7.55	8.42	10.40
Rice straw, mature	10.65	31.03	29.90	9.19	14.77
Tall-fescue hay, mature	18.93			17.51	11.93
Alfalfa, hay	24.43	24.02	26.20	33.00	31.75
**Chemical Composition, % of DM ^1^**	**CP 12.0%**	**CP 13.5%**	**CP 15.0%**	**CP 16.5%**	**CP 18.0%**
CP	12.00	13.50	15.00	16.50	18.00
A	0.72	0.85	0.98	1.21	1.37
B1	0.80	0.90	0.98	1.10	1.26
B2	8.00	9.00	10.00	11.00	12.00
B3	1.91	2.20	2.51	2.62	2.72
C	0.58	0.64	0.71	0.71	0.83
NDIP	2.49	2.84	3.22	3.32	3.54
ADIP	0.58	0.64	0.71	0.71	0.83
NDF	37.80	37.30	37.60	37.00	37.00
ADF	20.10	19.80	20.00	20.80	20.90
ADL	2.91	3.25	3.44	3.53	3.85
EE	2.23	2.21	2.07	1.04	1.05
NFC	43.90	43.00	41.50	41.80	40.30
Ash	6.55	6.86	7.03	6.97	7.17
Ca	0.48	0.52	0.55	0.60	0.60
P	0.28	0.26	0.26	0.23	0.23
TDN, % ^2^	70.30	69.80	69.50	69.10	68.60
tdNFC	43.00	42.10	40.90	41.80	40.30
tdCP	11.40	13.00	14.50	15.90	17.40
tdFA	1.31	1.21	1.09	0.16	0.16
tdNDF	19.90	19.00	18.80	18.10	17.70
DE (Mcal/kg) ^3^	3.11	3.11	3.12	3.12	3.12
ME (Mcal/kg) ^4^	2.69	2.69	2.70	2.70	2.70

^1^ CP: Crude protein; A: protein A fraction represents the soluble nonprotein N. Degradable rate in rumen is direct; B1: protein B1 fraction is the soluble true protein. Degradable rate in rumen is 120–400%/h; B2: protein B2 fraction represents protein with intermediate rates of degradation. Degradable rate in rumen is 3–16%/h; B3: protein B3 fraction is the CP insoluble in neutral detergent solution but soluble in acid detergent solution. Degradable rate in rumen is 0.06–0.55%/h; C: protein C fraction is the unavailable nitrogen [10,19]; NDIP: CP insoluble in neutral detergent solution; ADIP: CP insoluble in acid detergent solution; NDF: neutral detergent fiber; ADF: acid detergent fiber; ADL: acid detergent lignin; EE: ether extract; NFC: non-fiber carbohydrates. ^2^ True digestibility nutrition (%) = true digestibility non-fiber carbohydrate (tdNFC) + true digestibility crude protein (tdCP) + true digestibility fatty acid (tdFA) × 2.25 + true digestibility neutral detergent fiber (tdNDF) − 7 [11]. ^3^ Digestible energy (DE) = (tdNFC/100) × 4.2 + (tdCP/100) × 5.6 + (tdFA/100) × 9.4 − 0.3) [11]. ^4^ If EE was lower than 3%, metabolizable energy (ME) was (1.01 × DE) − 0.45, if EE was higher than 3%, ME was ((1.01 × DE) − 0.45) + 0.0046 × (EE − 3) [11].

**Table 3:** The chemical compositions of the feed ingredients used in trial 2.

Items ^1^ (% of DM)	Corn Grain, Ground, Dry	Wheat	Soybean Meal	Distillers Grains with Soluble, Dry	Rapeseed Meal	Corn Gluten Feed, Dry	Corn Gluten Meal, Dry	Tall-Fescue Hay, Mature	Alfalfa, Hay
CP	7.34	11.7	44.8	29.0	37.7	22.3	66.0	7.97	13.3
A	0.28	1.28	2.78	1.92	5.74	3.25	2.03	0.00	0.95
B1	0.81	1.43	1.03	1.39	6.77	8.88	2.00	1.29	0.19
B2	5.20	6.05	37.65	16.84	17.58	3.25	53.37	4.10	9.54
B3	0.93	2.79	3.16	8.07	0.00	6.42	8.57	0.69	2.14
C	0.13	0.11	0.15	0.76	7.62	0.53	0.00	1.89	0.50
NDIP	1.06	2.89	3.31	8.84	7.62	6.95	8.57	2.58	2.64
ADIP	0.13	0.11	0.15	0.76	7.62	0.53	0.00	1.89	0.50
NDF	11.09	15.87	12.61	41.01	28.58	40.10	21.62	65.27	40.53
ADF	3.03	3.65	7.60	16.1	19.8	10.4	2.68	41.4	25.2
ADL	0.41	0.39	0.59	1.38	16.9	2.97	1.05	4.70	5.06
EE	3.11	1.39	1.47	7.72	1.08	1.73	2.20	0.22	1.60
NFC	78.60	72.57	38.31	26.44	32.36	36.36	16.79	19.66	40.95
Ash	0.92	1.40	6.15	4.68	7.88	6.44	2.00	9.46	6.23
TDN ^2^	80.63	81.30	78.12	87.12	65.50	77.02	83.49	48.62	63.25
tdNFC	77.02	71.12	37.55	25.91	31.71	35.63	16.45	19.26	40.13
tdCP	7.29	11.62	44.71	28.68	34.67	22.11	65.96	6.00	12.74
tdFA	2.11	0.39	0.47	6.72	0.08	0.73	1.20	0.00	0.60
tdNDF	6.36	8.52	5.49	20.3	0.40	18.11	7.32	35.77	18.20
DE(Mcal/kg) ^3^	3.81	3.73	4.06	3.88	3.00	3.26	4.50	2.35	2.92
ME(Mcal/kg) ^4^	3.14	3.17	3.03	3.43	2.47	2.98	3.27	1.66	2.31

^1^ CP: Crude protein; A: protein A fraction represents the soluble nonprotein N. Degradable rate in rumen is direct; B1: protein B1 fraction is the soluble true protein. Degradable rate in rumen is 120–400%/h; B2: protein B2 fraction represents protein with intermediate rates of degradation. Degradable rate in rumen is 3–16%/h; B3: protein B3 fraction is the CP insoluble in neutral detergent solution but soluble in acid detergent solution. Degradable rate in rumen is 0.06–0.55%/h; C: protein C fraction is the unavailable nitrogen [10,19]; NDIP: CP insoluble in neutral detergent solution; ADIP: CP insoluble in acid detergent solution; NDF: neutral detergent fiber; ADF: acid detergent fiber; ADL: acid detergent lignin; EE: ether extract; NFC: non-fiber carbohydrates. ^2^ True digestibility nutrition (%) = true digestibility non-fiber carbohydrate (tdNFC) + true digestibility crude protein (tdCP) + true digestibility fatty acid (tdFA) × 2.25 + true digestibility neutral detergent fiber (tdNDF) − 7 [11]. ^3^ Digestible energy (DE) = (tdNFC/100) × 4.2 + (tdCP/100) × 5.6 + (tdFA/100) × 9.4 − 0.3) [11].^4^ If EE was lower than 3%, metabolizable energy (ME) was (1.01 × DE) − 0.45, if EE was higher than 3%, ME was ((1.01 × DE) − 0.45) + 0.0046 × (EE − 3) [11].

**Table 4:** The feed ingredients and chemical compositions used in trial 2.

Ingredient (%)	ME 2.4	ME 2.5	ME 2.6	ME 2.7	ME 2.8
Corn grain, ground, dry	10.00	15.18	20.18	25.74	31.29
Wheat	10.00	13.00	16.00	19.25	22.50
Soybean Meal, solvent, 44% CP	8.24	8.80	9.60	10.30	10.99
Distiller grains with soluble, dried		0.30	0.60	0.94	1.28
Rapeseed meal	5.42	5.50	6.00	6.79	7.59
Corn gluten feed, dry		0.70	1.43	0.71	
Corn gluten meal, dry	5.00	4.77	4.14	3.72	3.31
Tall-fescue hay, mature	30.00	26.75	23.05	19.27	15.48
Alfalfa, hay	31.34	25.00	19.00	13.28	7.56
**Chemical Composition, % of DM ^1^**	**ME 2.4**	**ME 2.5**	**ME 2.6**	**ME 2.7**	**ME 2.8**
CP	17.50	17.50	17.50	17.50	17.50
A	1.10	1.13	1.20	1.23	1.28
B1	1.22	1.33	1.45	1.48	1.50
B2	12.07	11.96	11.80	11.80	11.80
B3	1.94	1.98	2.00	1.96	1.92
C	1.17	1.10	1.06	1.03	1.00
NDIP	3.11	3.08	3.05	2.99	2.92
ADIP	1.17	1.10	1.06	1.03	1.00
NDF	38.65	35.45	32.16	28.60	25.01
ADF	22.81	20.30	17.79	15.24	12.70
ADL	4.09	3.69	3.36	3.04	2.73
EE	1.31	1.44	1.57	1.71	1.86
NFC	39.60	43.15	46.78	50.70	54.62
Ash	6.06	5.54	5.04	4.48	3.94
Ca	0.21	0.19	0.18	0.17	0.16
P	0.66	0.68	0.72	0.78	0.84
TDN ^2^	68.33	70.61	72.84	75.22	77.56
tdNFC	38.81	42.28	45.84	49.68	53.53
tdCP	16.54	16.64	16.72	16.80	16.88
tdFA	0.54	0.65	0.75	0.87	0.98
tdNDF	18.76	17.23	15.58	13.79	11.95
DE (Mcal/kg) ^3^	3.10	3.19	3.29	3.39	3.49
ME (Mcal/kg) ^4^	2.40	2.50	2.60	2.70	2.80

^1^ CP: Crude protein; A: protein A fraction represents the soluble nonprotein N. Degradable rate in rumen is direct; B1: protein B1 fraction is the soluble true protein. Degradable rate in rumen is 120–400%/h; B2: protein B2 fraction represents protein with intermediate rates of degradation. Degradable rate in rumen is 3–16%/h; B3: protein B3 fraction is the CP insoluble in neutral detergent solution but soluble in acid detergent solution. Degradable rate in rumen is 0.06–0.55%/h; C: protein C fraction is the unavailable nitrogen [10,19]; NDIP: CP insoluble in neutral detergent solution; ADIP: CP insoluble in acid detergent solution; NDF: neutral detergent fiber; ADF: acid detergent fiber; ADL: acid detergent lignin; EE: ether extract; NFC: non-fiber carbohydrates. ^2^ True digestibility nutrition (%) = true digestibility non-fiber carbohydrate (tdNFC) + true digestibility crude protein (tdCP) + true digestibility fatty acid (tdFA) × 2.25 + true digestibility neutral detergent fiber (tdNDF) − 7 [11]. ^3^ Digestible energy (DE) = (tdNFC/100) × 4.2 + (tdCP/100) × 5.6 + (tdFA/100) × 9.4 − 0.3) [11]. ^4^ If EE was lower than 3%, metabolizable energy (ME) was (1.01 × DE) − 0.45, if EE was higher than 3%, ME was ((1.01 × DE) − 0.45) + 0.0046 × (EE − 3) [11].

**Table 5:** The results of in vitro ruminal fermentation after 3, 6, 12, 24, and 48 h of incubations according to five protein levels (CP 12.0, 13.5, 15.0, 16.5, 18.0% of DM basis) and two incubation temperatures (39 and 41 °C).

Items ^1^	39 °C					41 °C					SEM	*p*-Value ^2^		
CP %	12.0	13.5	15.0	16.5	18.0	12.0	13.5	15.0	16.5	18.0		CP	Tem	CP × Tem
Ruminal fermentation after 3 h														
pH	6.73	6.72	6.72	6.73	6.73	6.75	6.74	6.74	6.74	6.74	0.04	0.445	<0.001	0.937
NH_3_-N, mg/dL	43.17	41.96	43.35	49.74	47.82	47.57	44.57	45.68	49.07	53.45	2.46	0.001	0.011	0.377
TVFA, mM	30.74	30.67	31.73	33.54	32.76	38.29	38.43	41.68	43.98	45.10	5.46	0.777	0.004	0.983
C2, %	68.56	68.50	69.12	68.03	68.00	69.35	69.67	69.25	69.33	69.22	0.67	0.812	0.019	0.825
C3, %	18.29	18.29	17.16	18.93	18.85	18.06	17.81	18.11	18.25	18.42	0.71	0.168	0.537	0.377
iC4, %	0.88	0.82	0.90	0.95	0.92	0.88	0.84	0.84	0.87	0.88	0.16	0.875	0.476	0.959
C4, %	10.18	10.37	10.66	9.86	9.99	9.70	9.80	9.89	9.45	9.39	1.17	0.169	0.004	0.972
iC5, %	0.80	0.79	0.82	0.86	0.86	0.74	0.69	0.69	0.73	0.75	0.09	0.780	0.009	0.967
C5, %	1.29	1.24	1.34	1.37	1.38	1.28	1.18	1.23	1.36	1.35	0.09	0.281	0.412	0.965
BCFA, %	1.68	1.60	1.72	1.81	1.79	1.61	1.53	1.52	1.60	1.62	0.25	0.832	0.108	0.974
A:P	3.76	3.76	5.79	3.60	3.62	3.85	3.92	3.83	3.80	3.77	0.65	0.401	0.517	0.406
LAB, µg/mL	241.4	231.3	343.6	253.2	231.9	246.0	210.3	229.0	238.6	241.8	39.09	0.768	0.590	0.927
Ruminal fermentation after 6 h														
pH	6.69	6.69	6.69	6.68	6.68	6.69	6.70	6.69	6.69	6.68	0.05	0.498	0.418	0.847
NH_3_-N, mg/dL	42.08	41.04	42.28	48.19	49.64	39.06	37.52	40.93	49.07	50.63	2.93	<0.001	0.386	0.748
TVFA, mM	38.32	37.83	38.08	41.41	42.56	41.06	40.01	42.48	42.88	44.33	5.73	0.036	0.014	0.864
C2, %	68.22	68.19	67.60	67.65	67.94	67.73	68.01	67.18	67.74	67.36	0.64	0.184	0.117	0.809
C3, %	18.50	18.34	18.52	18.59	18.47	18.77	18.43	18.69	18.31	18.62	0.63	0.914	0.679	0.914
iC4, %	0.87	0.84	0.85	0.88	0.88	0.89	0.87	0.90	0.91	0.93	0.10	0.249	0.025	0.919
C4, %	10.37	10.71	10.98	10.43	10.38	10.59	10.78	11.16	10.78	10.65	1.12	0.164	0.181	0.985
iC5, %	0.75	0.71	0.73	0.79	0.76	0.75	0.72	0.76	0.77	0.81	0.05	0.016	0.291	0.476
C5, %	1.29	1.21	1.32	1.65	1.58	1.27	1.19	1.32	1.50	1.62	0.06	<0.001	0.296	0.310
BCFA, %	1.61	1.55	1.58	1.67	1.64	1.64	1.59	1.66	1.68	1.74	0.15	0.065	0.078	0.745
A:P	3.69	3.72	3.65	3.65	3.69	3.61	3.69	3.60	3.71	3.64	0.12	0.864	0.544	0.895
LAB, µg/mL	266.8	251.6	250.7	236.6	274.5	244.0	232.7	245.0	235.1	247.2	18.93	0.474	<0.001	0.862
Ruminal fermentation after 12 h														
pH	6.55	6.56	6.58	6.60	6.62	6.55	6.59	6.60	6.61	6.62	0.06	0.002	0.334	0.948
NH_3_-N, mg/dL	36.54	36.72	41.37	52.93	54.24	40.93	43.75	50.62	56.88	62.69	4.25	<0.001	<0.001	0.632
TVFA, mM	53.39	51.91	54.57	54.12	54.11	57.94	54.93	55.24	57.06	57.31	6.31	0.450	0.004	0.738
C2, %	63.96	64.02	65.06	66.53	66.45	64.10	64.20	64.73	66.20	66.54	0.48	<0.001	0.865	0.942
C3, %	21.73	21.08	20.17	18.84	18.79	21.55	20.82	20.09	18.56	18.48	1.02	<0.001	0.455	0.999
iC4, %	0.86	0.87	0.91	0.96	0.93	0.92	0.94	0.97	1.01	1.02	0.05	<0.001	<0.001	0.777
C4, %	11.52	12.12	11.85	11.31	11.45	11.40	12.05	12.08	11.92	11.54	0.98	0.380	0.551	0.871
iC5, %	0.75	0.76	0.78	0.84	0.85	0.81	0.81	0.84	0.87	0.89	0.02	<0.001	<0.001	0.739
C5, %	1.18	1.14	1.24	1.53	1.54	1.21	1.19	1.28	1.44	1.53	0.03	<0.001	0.606	0.063
BCFA, %	1.61	1.64	1.69	1.80	1.78	1.73	1.75	1.81	1.88	1.91	0.07	<0.001	<0.001	0.930
A:P	2.95	3.05	3.24	3.57	3.57	2.98	3.09	3.23	3.61	3.64	0.20	<0.001	0.618	0.997
LAB, µg/mL	315.1	307.7	323.4	298.4	285.4	216.7	220.5	210.2	191.3	196.5	71.49	0.952	0.339	1.000
Ruminal fermentation after 24 h														
pH	6.48	6.48	6.52	6.54	6.55	6.50	6.48	6.50	6.54	6.57	0.06	<0.001	0.606	0.472
NH_4_, mg/dL	70.07	67.34	73.07	81.56	83.02	88.42	81.99	85.36	92.27	96.40	9.65	<0.001	<0.001	0.704
TVFA, mM	70.19	68.05	72.52	74.64	79.99	74.03	70.50	69.65	68.01	72.13	6.66	0.565	0.401	0.524
C2, %	62.85	63.20	64.92	66.58	67.05	63.64	62.78	63.62	65.11	65.32	0.55	<0.001	0.026	0.163
C3, %	21.22	20.62	19.23	17.45	17.35	20.53	20.63	19.69	17.82	17.91	0.80	<0.001	0.574	0.514
iC4, %	1.20	1.18	1.14	1.16	1.12	1.29	1.27	1.27	1.28	1.33	0.05	0.736	<0.001	0.355
C4, %	12.21	12.57	12.35	12.27	12.03	11.84	12.67	12.76	13.03	12.58	0.96	0.481	0.258	0.653
iC5, %	1.13	1.10	1.04	1.03	0.99	1.22	1.22	1.19	1.17	1.21	0.04	0.114	<0.001	0.418
C5, %	1.39	1.33	1.33	1.50	1.45	1.47	1.44	1.47	1.58	1.65	0.06	0.001	<0.001	0.564
BCFA, %	2.33	2.28	2.17	2.19	2.11	2.51	2.49	2.46	2.46	2.54	0.08	0.390	<0.001	0.379
A:P	2.97	3.07	3.38	3.84	3.88	3.10	3.05	3.24	3.69	3.67	0.16	<0.001	0.215	0.470
LAB, µg/mL	225.3	202.2	178.2	185.4	192.2	149.5	152.6	153.2	141.0	150.8	68.18	0.991	<0.001	1.000
Ruminal fermentation after 48 h														
pH	6.48	6.50	6.48	6.52	6.52	6.49	6.48	6.49	6.56	6.55	0.07	0.003	0.197	0.463
NH3-N, mg/dL	110.28	114.58	116.44	127.20	137.57	137.22	129.18	130.78	135.89	145.89	15.29	0.026	0.002	0.579
TVFA, mM	104.81	95.69	111.33	105.71	105.39	84.66	84.78	83.99	84.07	85.38	11.39	0.974	0.010	0.964
C2, %	64.23	63.77	65.20	66.69	66.67	62.81	63.05	63.19	64.84	64.79	0.79	0.004	0.002	0.870
C3, %	20.07	19.73	18.85	17.05	17.05	20.32	19.89	19.33	17.43	17.37	0.62	<0.001	0.194	0.993
iC4, %	1.41	1.44	1.33	1.33	1.36	1.60	1.54	1.54	1.52	1.54	0.08	0.770	0.002	0.957
C4, %	11.34	12.05	11.87	12.09	12.05	11.86	12.27	12.69	12.97	12.97	0.98	0.179	0.016	0.890
iC5, %	1.41	1.47	1.30	1.27	1.29	1.65	1.59	1.56	1.50	1.52	0.10	0.340	0.001	0.948
C5, %	1.54	1.54	1.45	1.57	1.58	1.76	1.65	1.69	1.73	1.81	0.10	0.546	0.001	0.912
BCFA, %	2.82	2.91	2.63	2.60	2.65	3.25	3.13	3.09	3.02	3.06	0.18	0.516	0.001	0.948
A:P	3.20	3.23	3.46	3.93	3.92	3.10	3.17	3.27	3.75	3.75	0.13	<0.001	0.043	0.961
LAB, µg/mL	169.8	143.0	147.5	145.1	138.0	166.2	162.1	159.8	147.1	136.8	24.12	0.634	<0.001	0.996

^1^ C2, acetate (mol/100 mol); C3, propionate (mol/100 mol); iC4, *iso*-butyrate (mol/100 mol); C4, butyrate (mol/100 mol); iC5, *iso*-valerate (mol/100 mol); C5, valerate (mol/100 mol); BCFA, branched chain fatty acid (added iC4 and iC5, mol/100 mol); A:P, acetate to propionate ratio; TVFA, total volatile fatty acids; LAB, rumen liquid-associated bacteria. ^2^ Data were analyzed using the MIXED procedure of SAS as mixed model, including crude protein (CP), incubation temperature (Tem) and CP × Tem as fixed effects and incubation run as a random effect.

**Table 6:** The results of in vitro ruminal fermentation after 3, 6, 12, 24, and 48 h of incubations according to five metabolizable energy levels (2.4, 2.5, 2.6, 2.7, and 2.8 Mcal/kg of DM basis) and two incubation temperatures (39 and 41 °C).

Items ^1^	39 °C					41 °C					SEM	*p*-Value ^2^		
ME, Mcal/kg	2.4	2.5	2.6	2.7	2.8	2.4	2.5	2.6	2.7	2.8		Energy	Tem	Energy × Tem
Ruminal fermentation after 3 h														
pH	6.90 ^abc^	6.89 ^bc^	6.89 ^bc^	6.90 ^bc^	6.88 ^c^	6.91 ^abc^	6.89 ^bc^	6.92 ^ab^	6.91 ^abc^	6.93 ^a^	0.01	0.140	<0.001	0.011
NH_3_-N, mg/dL	62.63 ^cd^	59.75 ^d^	66.22 ^bcd^	68.50 ^bc^	66.71 ^bcd^	80.18 ^a^	77.87^a^	77.92 ^a^	77.53 ^a^	74.13 ^ab^	1.61	0.133	<0.001	0.009
TVFA, mM	25.77	25.85	25.61	27.71	24.17	28.70	29.19	29.83	29.41	29.36	1.41	0.670	<0.001	0.678
C2, %	69.80	69.64	69.28	69.27	69.16	69.55	69.99	69.69	68.90	69.14	0.41	0.141	0.898	0.666
C3, %	17.15	16.73	17.14	16.91	16.80	17.17	17.03	16.98	16.94	16.78	0.45	0.353	0.793	0.827
iC4, %	0.88	0.92	0.90	0.92	0.93	0.92	0.88	0.90	1.00	0.96	0.02	0.032	0.153	0.190
C4, %	10.12	10.66	10.71	10.78	10.98	10.21	10.06	10.35	10.82	10.88	0.23	0.003	0.110	0.291
iC5, %	0.88	0.90	0.87	0.92	0.91	0.91	0.86	0.90	1.01	0.96	0.03	0.019	0.105	0.163
C5, %	1.17	1.15	1.09	1.20	1.21	1.24	1.18	1.19	1.34	1.28	0.03	0.001	<0.001	0.525
BCFA, %	1.76	1.82	1.77	1.84	1.84	1.82	1.74	1.80	2.01	1.92	0.05	0.023	0.122	0.173
A:P	4.07	4.16	4.05	4.10	4.13	4.05	4.12	4.11	4.07	4.12	0.13	0.688	0.855	0.921
LAB, µg/mL	48.73 ^bc^	34.73 ^c^	47.59 ^bc^	49.21 ^bc^	75.05 ^a^	63.29 ^ab^	61.48 ^ab^	52.91 ^bc^	60.42 ^ab^	51.43 ^bc^	3.86	0.009	0.011	<0.001
Ruminal fermentation after 6 h														
pH	6.85	6.86	6.87	6.86	6.86	6.86	6.87	6.85	6.85	6.85	0.01	0.215	0.261	0.067
NH_3_-N, mg/dL	70.90	67.49	66.85	65.11	63.44	72.23	77.06	72.05	78.23	75.61	2.49	0.696	<0.001	0.137
TVFA, mM	29.94 ^d^	30.81 ^cd^	30.61 ^cd^	31.00 ^cd^	29.69 ^d^	33.22 ^bc^	31.28 ^cd^	35.95 ^a^	35.59 ^ab^	32.17 ^cd^	0.60	<0.001	<0.001	0.002
C2, %	68.67 ^ab^	68.59 ^ab^	68.27 ^abc^	68.14 ^abc^	67.18 ^bcd^	68.89 ^a^	68.54 ^ab^	66.91 ^cd^	65.72 ^d^	66.03 ^d^	0.32	<0.001	<0.001	0.003
C3, %	17.42 ^bc^	17.27 ^c^	17.42 ^bc^	17.74 ^bc^	18.00 ^abc^	17.43 ^bc^	17.32 ^c^	18.61 ^ab^	19.09 ^a^	18.35 ^abc^	0.33	0.001	0.002	0.036
iC4, %	0.93	0.95	0.94	0.89	0.97	0.92	0.95	0.90	0.93	0.96	0.03	0.213	0.632	0.715
C4, %	10.81	10.91	11.12	11.07	11.53	10.56	10.95	11.36	12.02	12.42	0.37	<0.001	0.016	0.053
iC5, %	0.89	0.92	0.92	0.86	0.95	0.87	0.92	0.87	0.91	0.93	0.03	0.263	0.736	0.592
C5, %	1.27	1.36	1.33	1.29	1.38	1.33	1.32	1.35	1.34	1.31	0.04	0.693	0.795	0.506
BCFA, %	1.82	1.87	1.86	1.75	1.92	1.79	1.87	1.77	1.84	1.89	0.07	0.251	0.684	0.651
A:P	3.94 ^a^	3.97 ^a^	3.92 ^a^	3.85 ^ab^	3.74 ^abc^	3.95 ^a^	3.96 ^a^	3.60 ^bc^	3.44 ^c^	3.60 ^bc^	0.07	<0.001	<0.001	0.012
LAB, µg/mL	57.29 ^b^	67.55 ^b^	69.70 ^b^	60.99 ^b^	56.36 ^b^	51.97 ^b^	101.60 ^a^	63.90 ^b^	70.15 ^b^	58.72 ^b^	7.25	0.001	0.091	0.025
Ruminal fermentation after 12 h														
pH	6.76 ^ab^	6.75 ^b^	6.76 ^b^	6.73 ^b^	6.72 ^b^	6.81 ^a^	6.76 ^b^	6.76 ^b^	6.72 ^b^	6.74 ^ab^	0.01	<0.001	0.059	0.044
NH_3_-N, mg/dL	70.14 ^bcd^	67.57 ^bcd^	54.66 ^e^	62.76 ^cde^	59.95 ^de^	85.83 ^a^	73.11 ^bc^	75.98 ^ab^	66.51 ^bcd^	69.38 ^bcd^	2.33	<0.001	<0.001	0.006
TVFA, mM	42.23 ^cde^	40.00 ^e^	41.67 ^de^	42.75 ^cde^	47.05 ^abcd^	51.37 ^a^	45.03 ^bcde^	48.14 ^abc^	49.43 ^ab^	46.19 ^abcd^	1.19	0.010	<0.001	0.006
C2, %	65.10	65.27	63.86	62.22	60.56	65.44	65.65	62.19	60.05	60.18	0.60	<0.001	0.066	0.120
C3, %	20.39	19.86	20.58	21.35	23.03	20.29	19.76	21.04	22.55	22.31	0.49	<0.001	0.595	0.274
iC4, % *	0.86	0.92	0.90	0.87	0.81	0.91	0.88	0.86	0.82	0.87	0.02	0.013	0.909	0.016
C4, %	11.53 ^c^	11.75 ^c^	12.40 ^bc^	13.35 ^ab^	13.46 ^ab^	11.17 ^c^	11.58 ^c^	13.71 ^a^	14.48 ^a^	14.43 ^a^	0.33	<0.001	0.002	0.010
iC5, % *	0.82	0.87	0.87	0.86	0.83	0.87	0.83	0.83	0.80	0.86	0.02	0.830	0.261	0.037
C5, %	1.31	1.33	1.40	1.35	1.31	1.31	1.31	1.36	1.31	1.35	0.04	0.359	0.632	0.760
BCFA, % *	1.68	1.79	1.77	1.73	1.64	1.79	1.71	1.69	1.61	1.72	0.04	0.221	0.509	0.024
A:P	3.20	3.29	3.10	2.92	2.64	3.23	3.33	2.96	2.67	2.70	0.09	<0.001	0.323	0.319
LAB, µg/mL	76.08	82.96	88.02	62.35	78.51	76.26	72.78	110.54	86.13	75.90	10.23	0.099	0.284	0.314
Ruminal fermentation after 24 h														
pH	6.69	6.68	6.64	6.62	6.62	6.70	6.67	6.66	6.62	6.61	0.01	<0.001	0.615	0.364
NH_3_-N, mg/dL	88.34	82.91	77.29	78.03	78.18	106.45	109.57	111.89	102.15	99.69	3.02	0.039	<0.001	0.112
TVFA, mM	54.13	52.25	61.60	60.99	57.46	56.20	62.77	67.31	63.67	68.29	2.18	0.002	<0.001	0.163
C2, %	62.77	62.62	59.97	58.35	58.20	63.81	63.37	59.33	58.50	57.64	1.00	<0.001	0.820	0.884
C3, %	20.76	20.61	22.81	23.46	23.43	20.18	20.70	23.13	23.28	22.82	0.70	<0.001	0.551	0.853
iC4, %	1.05	1.02	0.98	1.00	0.91	1.04	1.10	1.12	1.01	1.08	0.07	0.798	0.060	0.534
C4, %	12.83	13.29	13.80	14.66	15.06	12.43	12.30	13.80	14.67	15.72	0.68	0.002	0.735	0.808
iC5, %	1.01	0.98	0.99	0.99	0.95	1.02	1.05	1.09	1.03	1.15	0.07	0.974	0.060	0.632
C5, %	1.58	1.48	1.44	1.54	1.45	1.52	1.48	1.55	1.51	1.59	0.08	0.920	0.541	0.684
BCFA, %	2.06	2.00	1.97	1.99	1.85	2.05	2.15	2.20	2.04	2.23	0.13	0.980	0.059	0.594
A:P	3.02	3.04	2.65	2.49	2.49	3.16	3.06	2.58	2.52	2.54	0.11	<0.001	0.579	0.850
LAB, µg/mL	102.82	85.68	82.23	87.47	77.98	78.84	85.40	99.02	76.20	97.56	6.87	0.666	0.969	0.022
Ruminal fermentation after 48 h														
pH	6.66	6.66	6.63	6.58	6.59	6.69	6.67	6.61	6.59	6.59	0.01	<0.001	0.389	0.065
NH_3_-N, mg/dL	143.75	128.69	139.64	134.25	133.72	140.73	141.87	142.03	150.58	146.15	7.79	0.813	0.062	0.554
TVFA, mM	70.58	76.68	73.21	78.01	79.03	69.91	75.17	77.92	79.04	76.94	1.83	0.002	0.801	0.375
C2, %	62.47	62.31	60.76	58.62	57.78	63.78	60.97	58.90	57.49	56.39	0.64	<0.001	0.043	0.150
C3, %	20.46	19.97	20.79	22.06	22.18	19.68	20.54	21.54	22.28	22.81	0.45	<0.001	0.337	0.450
iC4, %	1.54	1.53	1.43	1.42	1.45	1.44	1.43	1.36	1.44	1.35	0.04	0.085	0.014	0.534
C4, %	12.12	12.82	13.69	14.49	15.10	11.78	13.63	14.71	15.15	15.70	0.52	<0.001	0.111	0.731
iC5, %	1.54	1.54	1.46	1.49	1.56	1.47	1.49	1.42	1.54	1.51	0.05	0.336	0.298	0.712
C5, %	1.87	1.83	1.87	1.91	1.94	1.84	1.94	2.06	2.10	2.24	0.08	0.074	0.009	0.409
BCFA, %	3.08	3.07	2.89	2.92	3.00	2.91	2.92	2.79	2.99	2.85	0.08	0.336	0.074	0.612
A:P	3.06	3.13	2.93	2.66	2.61	3.24	2.97	2.74	2.58	2.48	0.08	<0.001	0.164	0.170
LAB, µg/mL	137.98 ^bcd^	138.38 ^bcd^	168.73 ^ab^	152.25 ^abc^	186.90 ^a^	108.45 ^d^	104.26 ^d^	102.84 ^d^	109.74 ^cd^	98.19 ^d^	8.69	0.125	<0.001	0.014

^1^ C2, acetate (mol/100 mol); C3, propionate (mol/100 mol); iC4, iso-butyrate (mol/100 mol); C4, butyrate (mol/100 mol); iC5, iso-valerate (mol/100 mol); C5, valerate (mol/100 mol); BCFA, branched chain fatty acid (added iC4 and iC5, mol/100 mol); A:P, acetate to propionate ratio; TVFA, total volatile fatty acids; LAB, rumen liquid-associated bacteria. ^2^ Data were analyzed using the MIXED procedure of SAS as mixed model, including energy levels, incubation temperature and energy × incubation temperature (Tem) as fixed effects and incubation run as a random effect. ^a–e^ Indicate that the means of the interactions of energy levels and incubation temperature are significantly different. * Indicates when there is a statistically significant difference in interaction in the MIXED procedure, but no significant difference in post-test Tukey.

## Data Availability

The data presented in this study are available in this paper.
